# The Yin–Yang Definition Model of Mental Health: The Mental Health Definition in Chinese Culture

**DOI:** 10.3389/fpsyg.2022.832076

**Published:** 2022-03-24

**Authors:** Kai Wang

**Affiliations:** Institute of Traditional Chinese Culture, Ocean University of China, Qingdao, China

**Keywords:** mental health, definition model, Confucianism, Taoism, Chinese culture

## Abstract

It is a common aim of psychologists to construct a definition model with universal cultural applicability for mental health. These models can be divided into two types in terms of definition: One is the negative mental health definition model based on the absence of mental illness symptoms; the other is the definition model of positive mental health based on subjective feelings, such as happiness and social identity. However, neither of these definitions can properly explain Chinese people’s understanding of mental health or how mental health is dealt with in Chinese culture. This paper proposes a Yin–Yang definition model of mental health based on the theory of personality of Confucianism and Taoism. This model not only properly describes the understanding of mental health in traditional Chinese culture, but also explains East Asian psychotherapy and mental health practices in the context of Chinese culture.

## Introduction

Although the term mental health was used in Europe as early as the mid-19th century (Rosen, 2015), until recently, mental health has lacked a clear and universal definition (Bertolote, 2008; Galderisi et al., 2015). In recent decades, as the WHO definition of mental health has been widely used in the academic world, many psychologists have devoted themselves to constructing a universal mental health definition model. With the development of cross-cultural psychology, psychologists have found it difficult to build a model of mental health that can be universally applied to all cultural communities (Galderisi et al., 2015; Zhaoyan, 2017). With this in mind, they have set out to find models of mental health based on different cultural traditions (Kuo and Kavanagh, 1994; Office of the Surgeon General [US], Center for Mental Health Services [US], and National Institute of Mental Health [US]., 2001; Leslie, 2002; Bass et al., 2007). These models can be divided into two types according to their cultural backgrounds: One is the individually centered definition model of mental health based on Western culture; the other is the non-individually centered mental health model based on Eastern culture. The former is built on egoism, emphasizing the individual’s normal psychological state or happiness maintained by satisfying individual desires; the latter, based on Confucian morals, equates mental health with a sense of morality. Both models have limitations: They cannot properly explain the Yin–Yang model, which is constructed based on Confucian moralism and Taoist liberalism in the context of traditional Chinese culture.

## Individually Centered Definition Models of Mental Health and Their Limitations

The exploration of an individual’s mental functions has always been an important issue in Western philosophy. The early philosophers represented by Plato used the soul to explain the uniqueness of human mental functions (Seeskin, 2008). With the development of natural science in modern times, Hume and other philosophers began to distinguish the mental and physical systems into two different entities from the perspective of empiricism and began to explore their structures (Flage, 1982). In the 19th century, psychology as a science formally became independent from philosophy. While continuing philosophers’ discussion of an individual’s mental structure and other issues, psychologists began to devote themselves to constructing a universally applicable mental health definition model under the influence of modern science. However, because scientism has not eliminated the influence of the long-standing humanist trend of thought on psychology, there have been two research directions in the construction of individually centered mental health models: one is the negative mental health model based on scientism, and the other is the positive mental health model based on humanism (Shuang and Minli, 2017).

### Negative Definitions of Mental Health

Since Hume, Western philosophical scholarship has generally paid attention to this problem: any form of mental expression can be naturalized into the “experience world,” and mental forms can be explained by the attributes and relationships that people find in the experience world (Cottrell, 2018). This explanation of mental functions that emphasizes experience causes modern psychologists to pay more attention to the empirical analysis of an individual’s mental state with empirical methods. Under this theoretical background, when Clifford Whittingham Beers, the first advocate of mental health suffering from mental illness, launched a mental health campaign in the United States aimed at seeking individual mental health by eliminating mental illness, scientism-based mental health definition models emerged. In these models, the basic principle of judging whether an individual has mental health issues is checking for the presence or absence of mental illness symptoms. These models take the empirical analysis of mental illness as being sufficient. They regard non-illness as health and demonstrate mental health from the perspective of mental illness. Therefore, academic circles call these models negative mental health definition models.

At first, the negative definitions of mental illness relied on biological explanations. Mental health was considered to encompass diseases of the brain, such as advanced syphilis or poison-induced psychosis (Szasz, 1960). Some argue that treating mental illness as a general illness would avoid the stigma of associating mental illness with moral anomie and mental disorders (Weiner, 1995). However, many psychologists are dissatisfied with this approach. They believe that the physiological definition of mental illness brings three types of negative consequences: First, it completely equates mental illnesses with physical illnesses, and does not show the unique characteristics of mental illnesses (Corrigan and Watson, 2004). Second, equating mental illnesses with brain defects could result in people forming the idea that mental illnesses are difficult to cure and thus could encourage social prejudice (Corrigan et al., 1999). Third, attributing mental illness to a physiological difference could lead to the public marginalizing people with mental illnesses as a distinct minority of the human race (Mehta and Farina, 1997; Phelan, 2002).

Since it was considered inadvisable to think of mental illnesses as diseases of the brain, psychologists began to think about the differences between mental and physical illnesses. The subsequent models can be divided into two categories: The first pins mental health on personality, putting a strong emphasis on the importance of social/personality development (Jahoda, 2013). The second considers both cognitive disorders and unusual behavioral patterns to be causes of poor mental health (Clausen, 1956). Although these two methods of thinking about mental illness and mental health have overcome the shortcomings of biological definition methods, their accuracy for judging mental illness has been questioned because they rely on objective psychological lists and scales. Scott pointed out that psychological lists and scales have limitations in setting standards, and it is easy to be judged as mentally ill by one scale and healthy by another scale. In addition, if psychological lists and scales are the determining factors in diagnosis, subjects can easily perceive this and manipulate the results according to their own will (Scott, 1958).

To overcome the negative impact of personal intentions on the diagnostic accuracy of mental health scales, psychologists began to explore a mental illness evaluation standard combining expert clinical diagnosis and personal intentions to seek medical treatment. Paul b. Lieberman, M.D., advocated combining objective methods with subjective experience to investigate individual psychological states. He proposed that expression and interpretation methods should be used instead of purely objectifying approaches, such as psychological scales. Using an expression and interpretation method means that the clinician should ask the patient to describe his/her mental state and then objectively analyze what he/she has said. The advantage of this approach is that the patient can no longer be limited by the scale and can express all of the factors related to his/her psychological state, such as personal behaviors, the situations in which the illness occurs, the common belief of his/her community, and his/her past. This allows the psychologist to gain a holistic view of the patient to make the most accurate clinical diagnosis (Lieberman, 1989). Unfortunately, this approach still fails to solve the problem of diagnosing mental illnesses posed by the Rosenhan experiment. In 1973, Rosenhan published an article describing an experiment in which eight mentally healthy people visited several public hospitals and one private hospital pretending to have mental illnesses. Nearly all of them were diagnosed with schizophrenia (Rosenhan, 1973). This is cause for psychologists’ ability to diagnose mental illnesses to be strongly questioned (Resnick, 1984; Wilson and Plumly, 1984; Moncrieff, 2010; Jabr, 2012).

To sum up, due to the uncertainties in diagnosing mental illnesses, psychologists cannot reach a unified standard for judging individual mental states from the perspective of mental illness. More importantly, since Goffman, psychologists have found that the public prejudice against mental illness is one of the important reasons for increasing the stringency of mental illness diagnostics: Once the individual is diagnosed with a mental illness, they often will be discriminated against, rejected, or looked down upon, so they may not want to admit their bad mental state (Goffman, 1963; Byrne, 2000). The emergence of this phenomenon made psychologists realize that it is not a good choice to define mental health from the negative perspective of disease, which made it possible for positive psychologists to explore the definitions of mental health from the perspective of humanism.

### Positive Definitions of Mental Health

It is difficult for psychologists to form a unified definition of mental illness because of the difficulties in diagnoses, and it is easy to stigmatize mental illnesses by talking about them from the perspective of illness. Therefore, since World War II, psychologists have sought to replace the negative mental health definition focusing on mental illness with a positive mental health definition focusing on health (Pilgrim, 2009). Psychologists generally recognize that mental health is the ability of an individual to maintain his or her health, but have different ideas about what that ability is.

Of course, it should be noted that the above analysis is not intended to show that the negative and positive definitions of mental health are opposed. They are just different ways of describing the psychological characteristics that a mentally healthy person should have. Complete mental health includes both negative mental health and positive mental health (Schnfeld et al., 2017). From this point of view, the negative mental health definitions state that a mentally healthy person should be a person without mental illness, and the positive mental health definitions state that a mentally healthy person should have self-actualization, well-being, and meaning in life. They are not contradictory, but instead have a similar theoretical pursuit—that is, to help each individual construct a perfect self, a happy self (Træen et al., 2019; Bowins, 2021). However, positive mental health models, like negative models, have some limitations.

In 1951, the World Health Organization described mental health as an individual’s ability to establish good interpersonal relationships with others and to actively adapt to changes in the natural and social environment (WHO, 1951). This indicates the popularity of defining mental health in terms of individual ability. By 1958, there were so many of these theories that Jahoda wrote a book summarizing and analyzing them. Jahoda divided these theories into six categories (Jahoda, 1958), all of which aim at building or maintaining a perfect self. The first category of theories hold that mental health is when individuals can face themselves with a good attitude. The second kind of theory holds that mental health is the ability of individuals to be unique and achieve good self-growth and development. The third theory holds that mental health is the individual’s ability to integrate various excellent psychological qualities. The fourth theory holds that mental health is the ability of individuals to maintain autonomy in the face of social influences on themselves. The fifth theory holds that mental health is an individual’s ability to understand reality. The sixth theory asserts that mental health is an individual’s ability to control his/her environment. It is not difficult to see that the first three types of theories define mental health from the perspective of the individuals themselves, while the last three types of theories define health from the perspective of the relationship between individuals and reality. In addition to these definitions, there are some other comprehensive definitions. For example, Martin Seligman, who studies mental health based on “authentic happiness,” believes that a happy person should pursue a pleasant, engaged, and meaningful life (Seligman, 2002). Another Japanese term for mental health, Ikigai, suggests that healthy people have a sense of happiness, worth, and gratitude for being alive (Trudel-Fitzgerald et al., 2021). However, there are two limitations in all these definitions: One is that there is great uncertainty in the criteria for judging whether an individual has these abilities. Another limitation is that these definitions are built on an individually centered cultural context, which makes it difficult to explain the psychological state of people living in non-individually centered cultures. Let us examine these two limitations in detail.

First, in terms of evaluation criteria, to judge whether individuals can properly deal with the relationship between themselves and the environment, which requires individuals to act normally, it is necessary to judge what is normal and what is abnormal (Jahoda, 1958). The problem, as many scholars have realized, is that it is very difficult to define normal and abnormal. Take two of the most popular definitions of normal and abnormal, for example. We found that, first, the statistical definitions of normal and abnormal regard frequently occurring behaviors as normal behaviors, while regard infrequent behaviors as abnormal. On the one hand, this definition does not answer the question—what is “frequent”? Indeed, what is “infrequent”? It is difficult to find universal and uniform answers to such questions in our complex reality. In addition, this definition does not have good cultural adaptability. People’s behaviors vary greatly among different cultural communities, and behaviors that occur frequently in one community may be rare in another (Scott, 1958; Rogers and Pilgrim, 2014). Second, psychologists who define “normal” by ideal notions regard “normal” as some positive psychological state, such as self-actualization and stress resistance (Jahoda, 1958). The problem with this definition is that these ideal notions are theoretical presuppositions, and only those willing to accept these presuppositions as positive mental states will see them as normal. This definition is ineffective for those who are unwilling to acknowledge these ideal notions (Rogers and Pilgrim, 2014).

As far as cultures are concerned, Narayan and Hurriyet pointed out that cultural differences have an impact on different aspects of mental health, including perceptions of health and illness, coping styles, and patterns of seeking treatment (Galderisi et al., 2015). As Murphy stated, the abovementioned definitions mainly reflect North American cultural values (Murphy, 1978), so their limitation is that they do not have satisfactory cross-cultural validity; that is, they cannot be universally applied to all cultural systems. Specifically, the definition of mental health in the context of North American culture emphasizes the establishment and perfection of the individual, which is quite different from the non-individually centered Eastern culture, which emphasizes the elimination of self.

Although Western academic circles have noticed the limitations of individualism in strengthening individuals since 1912 (Hogg and Williams, 2000), and have tried to get psychologists to examine individual self-cognition from the perspective of collectivism; no matter how hard social psychologists try, under the influence of American individualism and European humanism, it is generally believed that forming a holistic and consistent concept of oneself is the key to maintaining individual health. Allport proposed, “There is no psychology of groups which is not essentially and entirely a psychology of individuals” (Graumann, 1986). From this angle, the conception of self from the perspective of collectivism still relies on individual psychology. In this context, the Western academic definition of mental health has always been in favor of enhancing personal belonging, satisfaction, and happiness, which is greatly different from the Eastern culture that emphasizes self-sacrifice (Feng, 1933), self-elimination (Wang and Wang, 2020), or self-transformation (Aggarwal, 2019). Therefore, we cannot apply these definitions of mental health to Eastern cultural communities, and it has become a new task for psychologists to establish a definition of mental health with oriental cultural characteristics.

## Non-Individually Centered Definitions of Mental Health and Their Limitations

In recent years, due to the inapplicability of the Western definitions of mental health to other cultures, more and more psychologists began to explore definitions of mental health based on Eastern culture. These can be called “non-individually centered” definitions to show the self-deconstruction characteristic of Eastern cultures. Although these definitions are highly diverse, none explain mental health in the context of Confucian and Taoist integration in China. The following two representative models are used to prove this.

### The Manas Model of Mental Health

Many psychologists have realized that the Western definitions of mental health are based on the theoretical background of the mind–body dualism in Western philosophy (Bennett, 2007; Santoro et al., 2009; Thirunavukarasu, 2011)—for example, the above analysis of mental health as the absence of illness is focused on the mental health definition from the perspective of the body. Additionally, defining mental health in relation to character and psychological states is focused on the perspective of the mind. This separation of body and mind is not common in Eastern cultures. Based on this consideration, Thirunavukarasu proposed the Manas mental health definition model by using the concept of Manas in Indian culture.

**FIGURE 1:**
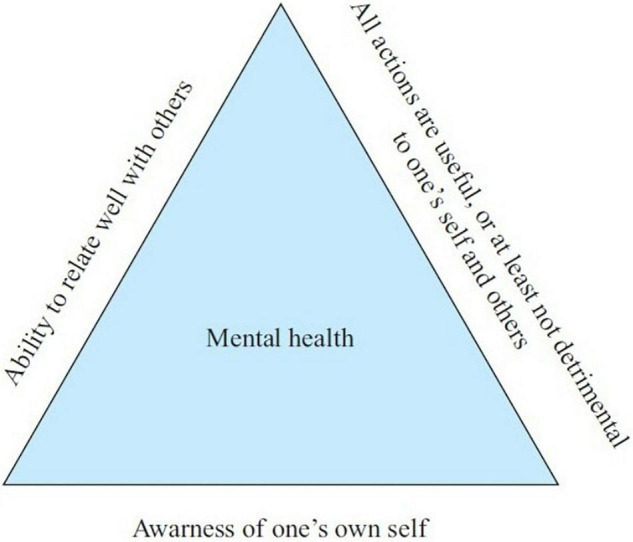
Triangular model of mental health.

Manas is a concept widely used in Hinduism and Buddhism. Although it is widely translated as the mind in modern English literature, it is quite different from the mind in Western philosophy. In Western philosophy, the mind is an entity different from the body, whereas Manas in Indian philosophy refers to a divine spiritual entity that transcends the opposition between body and mind. This entity can be regarded as a pure and divine cosmic soul, and the individual is the product of its interactions with the body and mind. Manas can be divided into thought, mood, and intellect, but each part is closely connected and indivisible. Individuals want to keep healthy to maintain the balance of these three. However, according to Manas, it is necessary to realize that individuals and others belong to the whole universe. Hence, one must construct the universal self and eliminate the individual self. On this basis, Thirunavukarasu constructed the triangle Manas mental health model (see Figure 1), indicating that mental health can be defined as the realization of the following three characteristics: (a) self-awareness (realizing that the self is not an individual, but a common Manas of human beings), (b) the ability to relate well with fellow human beings, and (c) understanding that all one’s deeds and activities are useful to oneself and others, or least not detrimental to oneself or others (Thirunavukarasu, 2011).

The limitation of the Manas model is that it is based entirely on Indian culture; therefore, the Manas model cannot explain the concept of mental health in different cultural contexts. Although Indian Buddhism was introduced into China, there is evidence that Buddhist beliefs are substantially altered in China. Some scholars have even put forward that Chinese Buddhism is not Buddhism (Stone, 1999). Therefore, this model certainly does not explain the concept of mental health in traditional Chinese culture.

### The Confucian and Taoist Models of Mental Health

In recent years, spirituality and religious coping have received widespread attention from psychologists, and some scholars have pointed out that religious coping strategies are effective in the treatment of mental illnesses. Considering this, Kam-Shing Yip proposed the influential Confucian and Taoist mental health models by analyzing traditional Chinese culture.

**FIGURE 2:**
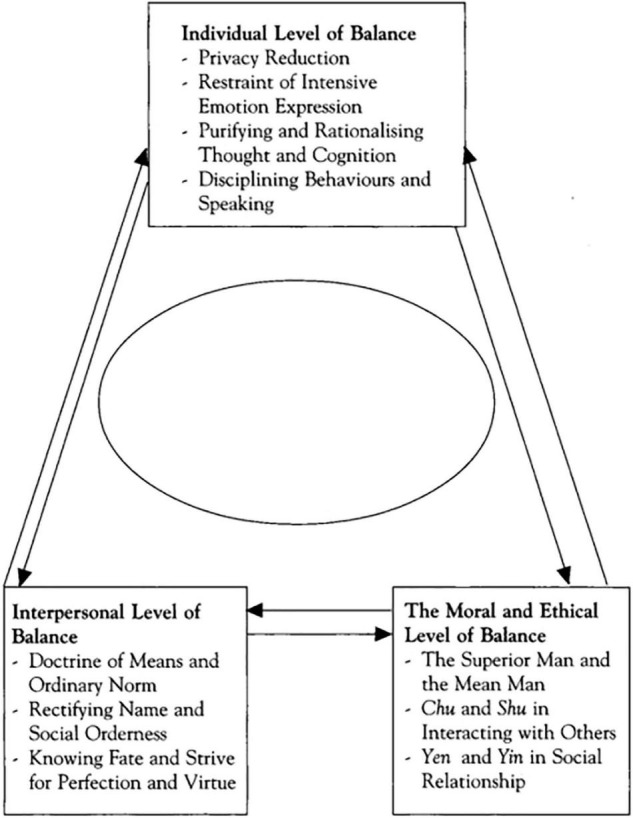
Confucian model of mental health.

**FIGURE 3:**
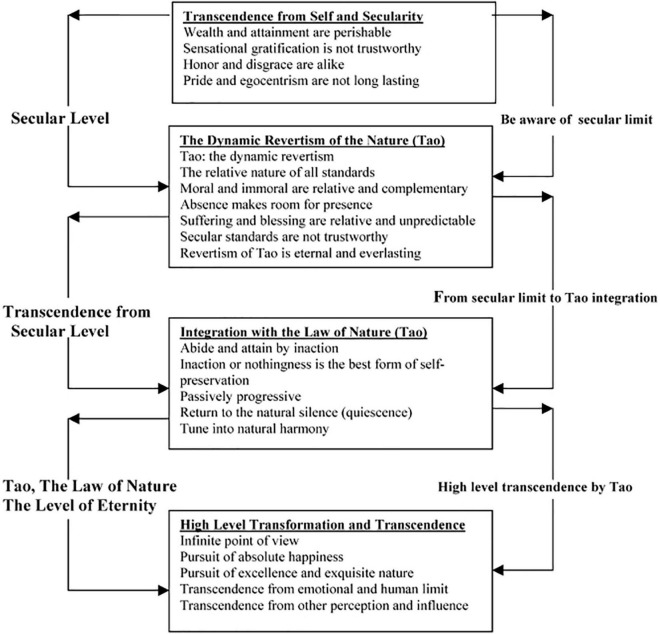
Mental health concept of classical Taoism by Lao Tzu and Chuang Tzu.

Based on the classic Four Books of Confucianism, Kam-Shing Yip defined the Confucian understanding of mental health as a direction that suggests self-discipline and obedience to social order to maintain one’s inner balance and external harmony with others. The three levels of harmony and balance are shown in a triangle model (see Figure 2). As the model indicates, to maintain mental health, individuals need to achieve three levels of balance—namely, the individual, interpersonal, and moral and ethical levels of balance (Yip, 2003). According to the viewpoints of Lao Zi and Zhuangzi, Kam-Shing Yip defined the Taoist understanding of mental health as the denial of self and the transcendence of individuals. Specifically, such transcendence is manifested in four aspects: First, the denial of the meaning of self-image and self-evaluation; second, going beyond one’s social attainments and entering into the laws of nature and having true inner peace; third, maintaining a state of inaction or “natural silence”; fourth, pursuing absolute and ultimate happiness rather than individual happiness. Based on these considerations, we can obtain a Taoist model of mental health (see Figure 3) (Yip, 2004).

These two models are very useful in explaining the concept of mental health held by Chinese people. However, the two models were constructed separately, yet in Chinese history, Confucianism and Taoism have always been closely combined and have had a joint cultural influence on Chinese people. The literature on which Kam-Shing Yip built the models mainly came from the pre-Qin period, which is insufficient to show the characteristics of Chinese culture after the Qin Dynasty. Therefore, these models cannot perfectly reflect the concept of mental health in traditional Chinese culture.

## The Yin–Yang Definition Model of Mental Health

### Individual Desires, the Law of Nature, and the Moral Norm

Although, generally speaking, people tend to divide traditional Chinese culture into Confucianism, Buddhism, and Taoism, there is ample evidence that Confucianism and Taoism were inseparable at each stage of development (Yanqing, 2002; Yu-xia, 2011; Cai and Geng, 2014). From the perspective of their origins, Confucius, the founder of Confucianism, and Lao Tzu, the founder of Taoism, are believed to have had contact. This is most well reflected in that Confucius once consulted Laozi. In addition, both early Confucianism and Taoism attached great importance to Zhouyi (*The Book of Changes*), and their ideological stances had many similarities. From the perspective of their development, although Confucianism and Taoism gradually developed in different directions after the pre-Qin dynasty, both Confucianism and Taoism still used Taiji diagrams to construct their theoretical systems until the Song Dynasty.

Based on Zhouyi (Zhenfu, 2018) and Taiji diagrams, which are valued by both Confucianism and Taoism, we can construct a Yin–Yang definition model of mental health with the common goal of eliminating individual desires.

Zhouyi is an early philosophical work in China. It expresses the basic views of Chinese people on the universe and life and has attracted much attention since its creation. Taiji and Yin–Yang are the basic concepts of Zhouyi. Taiji is the noumenon of the universe, and Yin and Yang respectively represent the static and dynamic states of Taiji. The static and dynamic changes of Taiji produce everything in the world (Ching, 1980; Baynes and Wilhelm, 2011).

**FIGURE 4:**
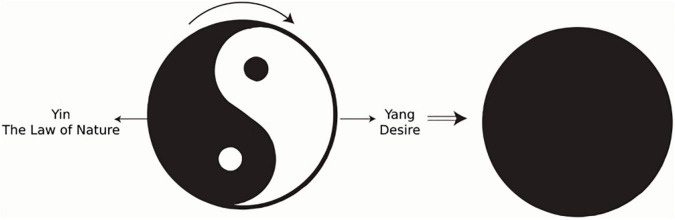
Taoist mental health definition model.

In terms of the interpretation of Taiji and Yin and Yang by Confucianism and Taoism, Taoism attaches importance to Yin, believing that Yin represents the law of nature and Yang represents the activities of people in pursuit of fame, wealth, and individual desires. Individual obedience to Yang’s activities will lead to the expansion of desires, creating a man-made world in opposition to nature, resulting in opposition between man and nature, bringing bondage to the individual’s mind. Meanwhile, if the individual obeys Yin, that is, the law of nature, he/she will eliminate the opposition between human and nature, so that the mind is free and mental health is sound. This view expressed in the model is embodied in Figure 4. In the figure, white represents Yang (desire) and black represents Yin (the law of nature). Tai Chi works clockwise, Yin into Yang: Personal desires are eliminated, Taiji becomes a harmonious whole, and individual freedom is united with nature. This provides mental health.

**FIGURE 5:**
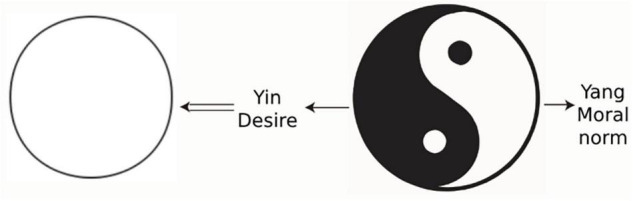
Confucian mental health definition model.

Confucianism values Yang and considers Yang to be the driving force for the formation of a good human society—the moral norm, whereas Yin is the individual’s pursuit of pleasure, fame, and wealth. An individual’s obedience toward Yin’s activities will lead to the expansion of desires, creating a selfish world that opposes society—a contradiction between the individual and the society—and bringing bondage to said individual’s mind. If an individual obeys Yang’s activities, he is in line with social ethics and actively seeks for the welfare of others, which will eliminate the contradiction between himself and society, free his mind, and maintain his mental health. This view is expressed in the model embodied in Figure 5, in which white areas represent Yang (the moral norm) and black areas represent Yin (personal desires). Taiji works clockwise, Yang into Yin: Personal desires are eliminated, Taiji becomes a harmonious whole, and the individual is unified with society; he has realized mental health.

### Confucian and Taoist Approaches to Mental Health

It can be seen from the Yin–Yang model that both Confucianism and Taoism regard individual desires as the fundamental cause of mental illness, so eliminating desires is the main way of achieving mental health. However, because Confucianism and Taoism focus on different aspects of Yin and Yang, and they have different approaches to eliminating desires.

**FIGURE 6:**
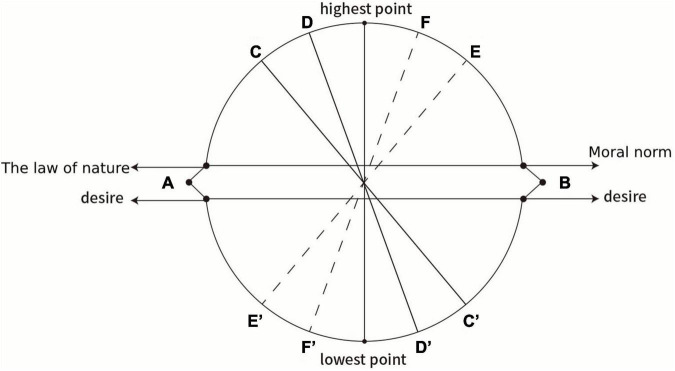
The changes in mental states of Confucianism and Taoism.

Specifically, Taoism emphasizes eliminating individual desires by bringing humans back to their natural state and integrating them with natural laws. According to the degree of desire elimination, humans can be divided into three types: Suren, Shanren, and Zhiren. Confucianism emphasizes suppressing selfish tendencies by transforming individual desires into collective desires. According to the degree of transformation, humans can be divided into the Xiaoren, Junzi, and Shengren types. The classifications of humans by Confucianism and Taoism is more clearly shown in Figure 6.

As far as Taoism is concerned, the first situation is that in the mind of a person, the power of natural law is weakest and the power of desire is strongest (both point A), and this person is Suren. A Suren is characterized by being smart and calculating, and he is bent on satisfying his desires without thinking about his relationship with nature and does not know that his desires deviate from natural laws. In the second case, in one’s mind, the force of natural law rises to point C and desire falls to point C’. At this point, the person can realize the importance of natural laws, but cannot completely control his/her desires, and this person is ordinary. In the third case, in one’s mind, the force of natural law rises to D and that of desire falls to D prime. At this point, that person is Shanren. He has a relatively good understanding of the natural law and can control his desires according to the natural law, but he still regards himself as having an independent existence, separate from nature. In the fourth case, in one’s mind, the force of natural law rises to its highest point and desire falls to its lowest point. This person is Zhiren. He regards himself as only a part of nature; to conform with nature is his principle for action.

In Confucianism, the first situation is that in the mind of a person, the powers of natural law and desire are both at point B: The power of moral norms is weakest, and the power of desires is strongest. This person is Xiaoren. Xiaoren is self-centered and focuses on satisfying his desires without thinking about his relationships with others. He does not know that his desires are not conducive to the development of society and his country. In the second case, the strength of the moral norms rises to point E and that of desires falls to point E’. At this point, the person can realize the importance of moral norms, but cannot completely control his/her desires. This person is ordinary. In the third case, moral strength rises to the F point and desires fall to point F’. In this case, the person is Junzi; he has a very good understanding of moral norms and can mostly control his desires to conform with moral norms, but he still makes mistakes. In the fourth case, the moral force rises to its highest point and desires fall to their lowest point in a person’s mind. In this case, that person is Shengren; he is characterized by the complete integration of himself with society as a whole, acting according to the needs of all mankind.

Both Taoism and Confucianism regard individual desires as the root of mental illness, and the integration of the individual with nature/society as a sign of mental health. Therefore, in real life, ordinary people need to improve themselves by practicing some approaches to eliminate or transform their desires to maintain mental health. The specific process is shown in Table 1.

**TABLE 1:** Confucian and Taoist approaches to mental health.

Taoism (Nature)				Confucianism (Society)			
	
Cultivation level	Types	The type of uncontrollable desire	Practice	Practice	The type of uncontrollable desire	Types	Cultivation level
High	Zhiren	None	1. Forget the difference and opposition between self and the universe	1. Benevolence	None	Shengren	High
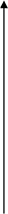	Shanren	Affinity for the law of nature	2. Keep mind empty and void	2. Righteousness	Affinity for the moral norm	Junzi	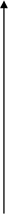
	Ordinary people	Love		3. Propriety	Acquisitiveness	Ordinary people		
Low	Suren	Clinging		4. Wisdom 5. Fidelity	Selfish	Xiaoren	Low

It is worth noting that in Confucianism and Taoism, the goal of eliminating desires is not to achieve a better self but to eliminate the self completely and make oneself part of society or nature, which is the concept of selflessness often mentioned in their writings (WEI Xindong, 2020). This idea is not only at odds with the Western definition of mental health, which aims to construct and perfect the self, but is also at odds with the existing Confucian and Taoist definitions of mental health, which only emphasize the elimination of individual desires.

## Conclusion

Mental health has lacked a clear and universal definition since the term was first used in Europe in the mid-19th century. According to the cultural ideas in question, the former definitions of mental health could be divided into two types: One is the individually centered definitions based on Western culture; the other is the non-individually centered definitions based on Eastern culture. The former type is based on egoism, emphasizing the realization of individual happiness by maintaining the unity of self and constructing a good self-image. The latter type, based on the Hindi, Confucian, and Taoist cultures, advocates the maintenance of a free psychological state by deconstructing the self and eliminating one’s own desires. Both models have their limitations and cannot correctly explain the Yin–Yang model based on Confucian moralism and Taoist liberalism in the context of traditional Chinese culture. Given this limitation, this study constructed a Yin–Yang mental health model based on Zhouyi and Taiji diagrams, which both Taoism and Confucianism attaching importance, using the historical fact that both Taoism and Confucianism influence Chinese people simultaneously. This model proposes, for the first time, that the basic view of Taoism and Confucianism on mental health is to eliminate or transform individual desires, and the specific way to eliminate or transform individual desires is to promote the unity of man with nature or society. This model not only establishes a mental health standard more in line with the actual thinking of Chinese people, but also helps to provide cultural resources for thinking about the psychological problems of modern Chinese youth.

## Author Contributions

The author confirms being the sole contributor of this work and has approved it for publication.

## Conflict of Interest

The author declares that the research was conducted in the absence of any commercial or financial relationships that could be construed as a potential conflict of interest.

## Publisher’s Note

All claims expressed in this article are solely those of the authors and do not necessarily represent those of their affiliated organizations, or those of the publisher, the editors and the reviewers. Any product that may be evaluated in this article, or claim that may be made by its manufacturer, is not guaranteed or endorsed by the publisher.
